# Serum cystatin C predicts the risk of non-ST-elevation acute coronary syndrome

**DOI:** 10.1186/s13019-023-02465-1

**Published:** 2023-12-01

**Authors:** Hao Dong, Dongping Xiao, Yong Tang

**Affiliations:** 1 Department of Cardiology, The Second Hospital of Nanjing, Nanjing University of Chinese Medicine, Nanjing, 210000 China; 2 Department of Cardiology, The First Hospital of Nanchang, Nanchang, 330000 China

**Keywords:** Non-ST-elevation acute coronary syndrome, Cystatin C, Inflammation, Biomarker

## Abstract

**Objective:**

Inflammation markers have been proposed as the predictors of adverse cardiac events in patients with non-ST-elevation acute coronary syndrome (NSTE-ACS). This study aimed to investigate prognostic value of serum cystatin C (Cys-C) for NSTE-ACS patients.

**Methods:**

Cys-C, neutrophil to lymphocyte ratio (NLR) and high-sensitivity C-reactive protein (hsCRP) were examined in 212 NSTE-ACS patients and 60 controls. Global registry of acute coronary events (GRACE) score and major adverse cardiac events (MACE) in NSTE-ACS patients were recorded.

**Results:**

Cys-C level in the serum was significantly higher in NSTE-ACS patients than in control, and was positively correlated with hsCRP level and NLR as well as GRACE score at admission and 6 months after discharge in NSTE-ACS patients. Serum Cys-C level was identified as a new predictor of MACE.

**Conclusion:**

Serum Cys-C level may be an inflammation biomarker in patients with NSTE-ACS, and could be used as an independent predictor of MACE.

## Introduction

Coronary artery disease (CAD) is a main cause of morbidity and mortality, and non-ST-elevation acute coronary syndrome (NSTE-ACS) is a common manifestation of CAD. During NSTE-ACS, atherosclerotic plaque initiates pathological processes, reduces coronary arterial blood supply and causes myocardial ischemia [1]. Global registry of acute coronary events (GRACE) score has been recommended to evaluate adverse outcomes in patients with acute coronary syndrome, and high GRACE score will increase the risk of death and help early intervention for high-risk patients to improve their prognosis [2].

Recent studies have shown that serum cystatin C (Cys-C) is closely associated with CAD [3, 4]. Cys-C was an independent predictor of cardiac events, and could be an indicator of CAD severity [5, 6]. Cys-C is also associated with an increased risk of death and could be used to predict major adverse cardiac events in patients with NSTE-ACS [7].

However, the relationship between serum Cys-C level and inflammation marker in NSTE-ACS patients is still unclear [8]. Therefore, in this study we aimed to analyze the relationship between Cys-C and inflammation marker in NST-ACS patients and evaluate the use of serum Cys-C level for early risk stratification of NST-ACS.

## Patients and methods

### Patients

This prospective study was approved by Ethics Committee of Nanjing University of Chinese Medicine (Approval No. 32,130) and all patients provided informed consent. This study recruited 212 NSTE-ACS patients (64.22 ± 14.35 years old) and 60 control participants (63.64 ± 8.01 years old) who proceeded coronary angiography to exclusive diagnosis of coronary heart disease. The two groups were recruited consecutively from January 2015 to May 2016 and matched for the age and gender. The inclusion criteria for NSTE-ACS group were: diagnosed as NSTE-ACS according to Guidelines for the diagnosis and treatment of NSTE-ACS; had complete medical data including history of disease and clinical and biochemical tests; willing to cooperate for follow-up. The exclusion criteria for NSTE-ACS group were: the patients had other heart diseases such as myocarditis diagnosed by magnetic resonance imaging, severe inflammatory diseases, serious hepatic and renal failure, anemia or cancer.

All patients were evaluated for routine blood examination, electrocardiogram and routine clinical laboratory tests, including the liver and kidney function tests, troponin and brain natriuretic peptide (BNP), total plasma cholesterol (TC), triglycerides (TG), high density lipoprotein cholesterol (HDL-C), creatinine (Cr), low density lipoprotein cholesterol (LDL-C), high-sensitivity C-reactive protein and Cys-C. Global registry of acute coronary events (GRACE) score were calculated from eight variables as described previously [9].

## Follow-up

Major Adverse Cardiac Events (MACE) were recorded for each patient during the follow-up of three months, including cardiac shock, recurrent myocardial infarction and angina, heart failure and any cause of death,

### Statistical analysis

Normally distributed data were presented as mean ± standard deviation. Abnormally distributed data were presented as median. All data were analyzed using SPSS version 17.0 (SPSS Inc, Chicago, IL, USA). Normally distributed data were compared by student *t* test and abnormally distributed data were compared by Wilcoxon test. Categorical variables were compared by Chi-square test. The relationship of indexes was analyzed by Pearson correlation analysis. A receiver operating characteristic (ROC) curve analysis was performed to calculate optimal cut-off value of Cys C for predicting MACE. P < 0.05 indicated statistical significance.

## Results

### Baseline characteristics of the subjects

We consecutively recruited 500 patients and 250 controls who proceeded coronary angiography to exclusive diagnosis of coronary heart disease. Finally, we included 212 patients and 60 control in NSTE-ACS and control groups, respectively. Among 212 patients in NSTE-ACS group, they were further qualified into unstable angina and non-ST elevation MI (NSTE-MI) according to troponin levels. About 98 patients (46.2%) were diagnosed as NSTE-MI. Baseline characteristics of the patients in NSTE-ACS and control groups showed no significant difference including the age and gender (Table 1).

**Table 1:** Baseline characteristics of NSTE-ACS patient and control groups

	NSTE-ACS group n = 212			Control group n = 60	*P*
Age, years Male gender, n (%) Smoking, n (%) Hypertension, n (%) Diabetes, n (%) BMI, kg/m^2^ Cr, umol/L TC, mmol/L TG, mmol/L HDL-C, mmol/L LDL-C, mmol/L hs-CRP, mg/L^#^ NLR^#^ Admission GRACE score Discharge GRACE score Cys-C, mg/L		64.23 ± 14.34 133 (62.74) 75 (35.38) 105 (49.53) 40 (18.87) 25.35 ± 5.36 65.59 ± 14.78 4.53 ± 1.25 1.44 ± 0.85 1.23 ± 0.32 3.29 ± 0.99 10.40 (6.63,26.20) 5.03 (3.47,9.56) 143.74 ± 44.94 111.32 ± 30.78 0.94 ± 0.28	63.64 ± 8.01 37 (61.67) 20 (33.33) 29 (48.33) 15 (25.00) 24.33 ± 2.81 63.53 ± 17.74 4.51 ± 1.08 1.37 ± 0.64 1.27 ± 0.22 3.07 ± 0.66 0.47 (0.16,0.85) 1.45 (1.21,1.92) - - 0.76 ± 0.12		0.83 0.88 0.77 0.87 0.30 0.40 0.57 0.95 0.67 0.46 0.25 0.00 ^*^ 0.00 ^*^ - - 0.00^*^

Value are mean ± SD except where expressed as median (quartile 1, quartile 3)^#^. *P < 0.05 compared to control group. *NSTE-ACS* Non-ST-elevation acute coronary syndrome; *BMI* Body mass index; *TC* Total plasma cholesterol; *TG* Triglycerides; *HDL-C* High density lipoprotein cholesterol; *LDL-C* Low density lipoprotein cholesterol; *hsCRP* High-sensitivity C-reactive protein; *NLR* Neutrophil to lymphocyte ratio; *GRACE* Global registry of acute coronary events; *Cys-c* Cystatin c

### Association of serum Cys-C level with characteristics of NSTE-ACS patients

As shown in Table 1, NSTE-ACS group and control group showed significant difference in serum Cys-C level (0.94 ± 0.28 vs. 0.76 ± 0.12 mg/L, *P* = 0.00). In addition, the two groups showed significance differences in hsCRP and NLR.

Among 212 NSTE-ACS patients, 135 patients (63.7%) received revascularization procedures such as percutaneous coronary intervention (PCI) or coronary artery bypass grafting (CABG) after admission. Serum Cys-C level showed positive correlation with GRACE score at admission and six months after discharge (*r* = 0.322, *P* = 0.030 and *r* = 0.394, *P* = 0.002, respectively). Serum Cys-C level also showed positive correlation with hsCRP and NLR (*r* = 0.404, *P* = 0.000 and *r* = 0.323, *P* = 0.003, respectively), and the age and Cr (*r* = 0.443, *P* = 0.000 and *r* = 0.662, *P* = 0.000, respectively). However, serum Cys-C level was not correlated significantly with TG, TC, LDL-C and HDL-C.

### Serum Cys-C level predicts MACE

**Fig. 1:**
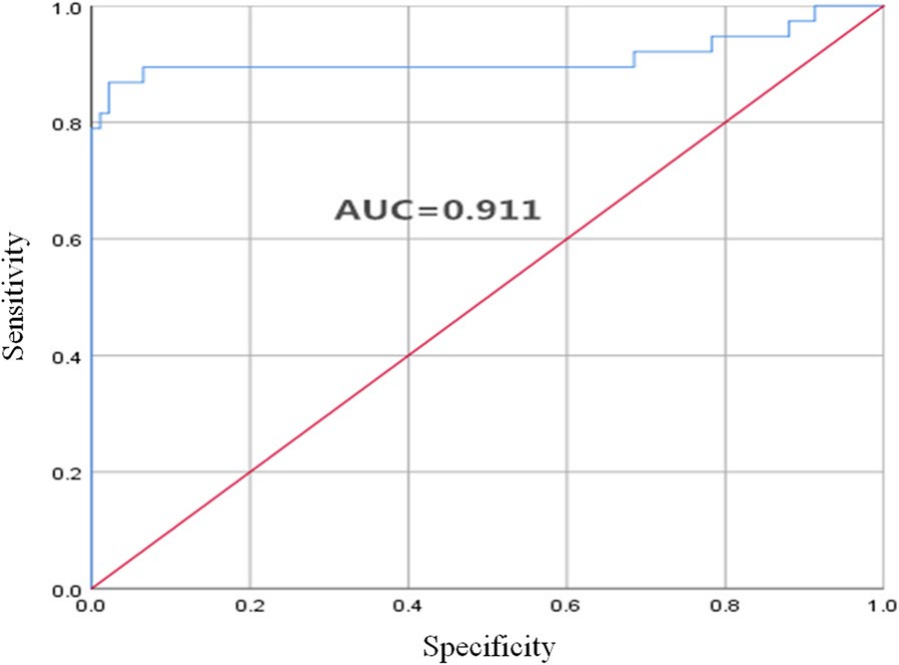
ROC curve analysis of the predication of MACE based on serum CHI3L1 level. Area under the curve (AUC) was 0.911

Logistic regression analysis using single factor showed that Cys-C [odds ratio (OR) 8.271, 95% confidence interval (CI) 2.670-25.624, *P* < 0.001], age (OR 1.050, 95% CI 1.025–1.076, *P* < 0.001), and TG (OR 0.647, 95% CI 0.448–0.936, *P* = 0.02) could be independent predictors of MACE (Table 2). Logistic regression analysis using multiple factors showed that Cys-C (OR 5.403, 95% CI 1.203–24.253, *P* = 0.03) could be an independent predictor of MACE. ROC curve analysis showed that for serum Cys-C level at cutoff value of 0.9 mg/L, the sensitivity was 86.1% and the specificity was 96.8% (Fig. 1).

**Table 2:** Logistic regression analysis of the factors predicting MACE in NSTE-ACS patients

Variables	OR	95% CI	*P* value
Cystatin C	8.271	2.670z–25.624	< 0.001
Age	1.050	1.025–1.076	< 0.001
Triglycerides	0.647	0.448–0.936	0.02

OR: odds ratio, 95% CI 95% Confidence interval

## Discussion

In this study, we demonstrated significantly higher serum Cys-C level in NSTE-ACS patients compared to controls. In addition, serum level of Cys-C was correlated with GRACE score at admission and after discharge in NSTE-ACS patients, and was also correlated with inflammation markers such as hsCRP and NLR. Finally, we identified Cys-C as an independent predictor of MACE.

Cys-C is an inhibitor of cysteine protease. Compared with the creatinine and urea nitrogen, Cys-C is less likely to be affected by gender, age, diet and other factors. The kidney is the only organ that eliminates serum Cys-C, thus serum Cys-C plays important role in early evaluation of renal insufficiency [10].

Serum Cys-C could predict the risk of heart failure, stroke and death in high-risk populations [3–5]. Cys-C may be involved in the pathogenesis of CAD by several mechanisms: (1) Cys-C could adjust the activity of cysteine protease, thus maintain dynamic balance of extracellular matrix, which is involved in the pathogenesis of CAD [11]. (2) Cys-C is actively involved in matrix remodeling associated with plaque regression, and the level of cystatin C is positively correlated with plaque area [12]. (3) During Cys-C oxidation, free radicals are generated to increase the formation of foam cells and induce artery luminal stenosis, leading to artery luminal stenosis. Cys-C also promotes the proliferation and migration of vascular smooth muscle cells [13, 14].

In this study we found that serum Cys-C level was high in NSTE-ACS patients. In addition, logistic regression analysis showed that serum Cys C level was the independent predictor of MACE, consistent with previous study [6]. Notably, we confirmed that Cys-C serum level was correlated with inflammation markers hsCRP and NLR, which are involved in coronary heart disease [15]. These findings suggest that Cys-C could affect the phagocytosis and chemotactic function of granulocyte, and participate in the process of inflammation to promote the pathogenesis of CAD.

Due to the high mortality and poor prognosis of NSTE-ACS, early risk stratification is essential to patients with NSTE-ACS. Various scoring systems have been developed for prognostic and risk stratification of NSTE-ACS patients. AHA and ESC guidelines emphasize the significance of GRACE score, and recommend it for routine use [3]. In-hospital GRACE score > 140 is considered as increased risk of mortality. In addition, recent studies have proposed a variety of parameters to predict the outcomes of ACS patients [16–19]. In this study, we found that serum Cys-C level was correlated positively with GRACE score at admission and after discharge, indicating that serum Cys-C level may be used for early risk stratification. Early detection and treatment of NSTE-ACS patients with high Cys-C level in the serum may improve the prognosis and reduce the mortality.

This study has several limitations. First, this study is a single-center observational study with possible bias for patient selection. Second, the sample size is relatively small. Third, we only measured several laboratory parameters and did not detect other parameters such as serum cortisol level [20]. Further large-scale multiple-center studies are needed to confirm our conclusion.

In conclusion, serum Cys-C may be involved in the progression of NSTE-ACS and become a useful biomarker of inflammation. Detection of serum Cys-C level may help early risk stratification to predict the prognosis of NSTE-ACS patients.

## Data Availability

The datasets used and/or analysed during the current study available from the corresponding author on reasonable request.
